# A Multi-Information Fusion Method for Gait Phase Classification in Lower Limb Rehabilitation Exoskeleton

**DOI:** 10.3389/fnbot.2021.692539

**Published:** 2021-10-29

**Authors:** Yuepeng Zhang, Guangzhong Cao, Ziqin Ling, WenZhou Li, Haoran Cheng, Binbin He, Shengbin Cao, Aibin Zhu

**Affiliations:** ^1^Guangdong Key Laboratory of Electromagnetic Control and Intelligent Robots, Shenzhen University, Shenzhen, China; ^2^Institute of Robotics and Intelligent Systems, Xi'an Jiaotong University, Xi'an, China

**Keywords:** sEMG, multi-information fusion, gait phase classification, lower limb rehabilitation exoskeleton, convolutional neural network (CNN), real-time

## Abstract

Gait phase classification is important for rehabilitation training in patients with lower extremity motor dysfunction. Classification accuracy of the gait phase also directly affects the effect and rehabilitation training cycle. In this article, a multiple information (multi-information) fusion method for gait phase classification in lower limb rehabilitation exoskeleton is proposed to improve the classification accuracy. The advantage of this method is that a multi-information acquisition system is constructed, and a variety of information directly related to gait movement is synchronously collected. Multi-information includes the surface electromyography (sEMG) signals of the human lower limb during the gait movement, the angle information of the knee joints, and the plantar pressure information. The acquired multi-information is processed and input into a modified convolutional neural network (CNN) model to classify the gait phase. The experiment of gait phase classification with multi-information is carried out under different speed conditions, and the experiment is analyzed to obtain higher accuracy. At the same time, the gait phase classification results of multi-information and single information are compared. The experimental results verify the effectiveness of the multi-information fusion method. In addition, the delay time of each sensor and model classification time is measured, which shows that the system has tremendous real-time performance.

## Introduction

Disability of the lower body or related body parts will lead to walking difficulties (Jung et al., 2015). Gait recovery is one of the main goals of patients with lower limb motor dysfunction (Wolbrecht et al., 2008). The traditional rehabilitation process is labor-intensive that several therapists are required throughout the training of one patient (Yang et al., 2019). The wearable lower limb rehabilitation exoskeleton is used for gait rehabilitation of patients with lower limb dysfunction (Yin et al., 2020; Céspedes et al., 2021), such as spinal cord injury, cerebral palsy, and stroke (Hobbs and Artemiadis, 2020; Nolan et al., 2020). A suitable lower limb rehabilitation exoskeleton will improve the life quality of patients with lower limb disorder greatly (Young and Ferris, 2017). In order to realize smooth human-machine coupling and achieve robot-facilitated rehabilitation training, it is necessary to synchronize the action of wearable lower limb rehabilitation exoskeleton with that of the body. Therefore, accurate classification of the gait phase is required. The classification of the gait phases correctly is critical for robots to assist timely (Wei et al., 2021).

The human leg sEMG signal can offer valuable motion information, such as symmetric and periodic motion in human gait (Deng et al., 2020; Gao et al., 2021; Yao et al., 2021), and it is characterized by simple signal acquisition, intuitive data, and the non-invasive acquisition method (Kim et al., 2018; Lin et al., 2020; Ma et al., 2020). Artificial neural networks have made great progress, are widely used in the field of classification, and have shown great performance (Adewuyi et al., 2016; Atzori et al., 2016). Therefore, sEMG of legs is combined with an artificial neural network (Cheng et al., 2020) in human gait phase classification (Lee et al., 2017). Morbidoni et al. (2019) proposed a deep learning method for classifying a swing phase and a stance phase. This method is mainly based on the sEMG signal and does not need to extract features from the signal. Through the test of 12 subjects, the accuracy is up to 92.6%, which proves the effectiveness of the sEMG signal in gait classification. Joshi et al. (2013) obtained sEMG data from human lower limbs and used the machine learning method to classify each stage of the gait cycle, which improved the classification accuracy of each stage of the gait cycle. Ziegier et al. (2018) proposed a method based on EMG data to classify the standing stage and the swing stage of the gait of healthy people by using bilateral leg muscle signals. This method introduces a new EMG feature, which is calculated according to the EMG of muscle pairs on both sides, and the classification accuracy of the proposed method reaches 96%. Di Nardo et al. (2021) studied the influence of different sEMG signal processing specifications and different numbers of sEMG sensors on the performance of the gait phase classification method based on neural network prediction and obtained an average accuracy of 93.4%. However, although the above methods have good performance, the accuracy still needs to be improved.

Plantar pressure is widely used in the research of gait phase classification (Joo et al., 2014; Xie et al., 2020). Luo et al. (2020) arranged plantar pressure sensors at the heel and toe and used the working state of plantar pressure sensors to classify gait stages, and the accuracy of this method reached 94.1%. Nazmi et al. (2019), respectively, arranged plantar pressure sensors under the heel and thumb, and divided the gait phase by analyzing the contact state between the heel and toe and the ground; the accuracy of this method reached 87.5 and 77%. Although the above method is enough to detect gait events, the accuracy of gait phase classification is not high, and the phase classification is relatively rough. In addition, Liu et al. (2016) used a single-joint angle to classify the gait phase, and the accuracy reached 94.45%, which proved the feasibility of the method. Grimmer et al. (2019) used the angle sensor to detect the stance and swing and obtained good results. However, the target achieved only by this method still needs to be improved.

Whether the above information can be fully combined to find out the accurate relationship to improve the accuracy of gait phase classification is an interesting problem. Therefore, a multi-information fusion method for gait phase classification in the lower limb rehabilitation exoskeleton is proposed to improve the classification accuracy in this article. Firstly, the gait phase classification experiments at different speeds were carried out and analyzed, and the accuracy of gait phase classification was significantly improved. Secondly, the gait phase classification results of multi-information and single information are compared. The experimental results show that the gait phase classification method based on multi-information fusion has good performance.

The structure of this paper is as follows: The second section introduces the gait phase classification system, gait information acquisition device, data preprocessing, and the neural network model for gait phase classification. The third section shows the design of the gait acquisition experiment and the software environment of the experiment. The fourth section is the result and discussion of the experiment.

## Methodology

### Gait Phase Classification System

A real-time gait classification system based on wireless multi-information fusion is designed and implemented. The gait classification system of human lower limb movement consists of the gait information acquisition part and the gait information processing part. The gait information acquisition part includes plantar pressure acquisition, knee angle acquisition, and sEMG signal acquisition. The acquired knee joint angle information and plantar pressure information are, respectively, transmitted to the single-chip microcomputer (Atmel atmega328p microprocessor, ATMEL Inc., USA) and two linear voltage modules (FRP resistance voltage converter, Telesky Inc., China). The output of the sensor that acquires the knee joint angle and plantar pressure is an analog quantity, and the single-chip microcomputer performs 50 Hz A/D sampling on it and records the time stamp at the same time. In the experiment, the microcomputer and the linear voltage module were integrated into an aluminum metal box with a length of 180 mm, a width of 160 mm, and thickness of 48 mm, and the aluminum was sealed and wrapped with tin foil to shield the interference of space clutter signals. The function of the linear voltage module is to convert the resistance signal of the thin-film pressure sensor into the voltage signal. The part of gait information processing is mainly a computer and the neural network model. The communication between the microcomputer and the linear voltage module and the computer uses the Lora wireless transmission module (Lora-01, Alientek Ltd, China) to transmit data information, which reduces the energy loss of wired transmission, and the redundancy of the connection line and is more convenient to wear and move at any time.

**Figure 1:**
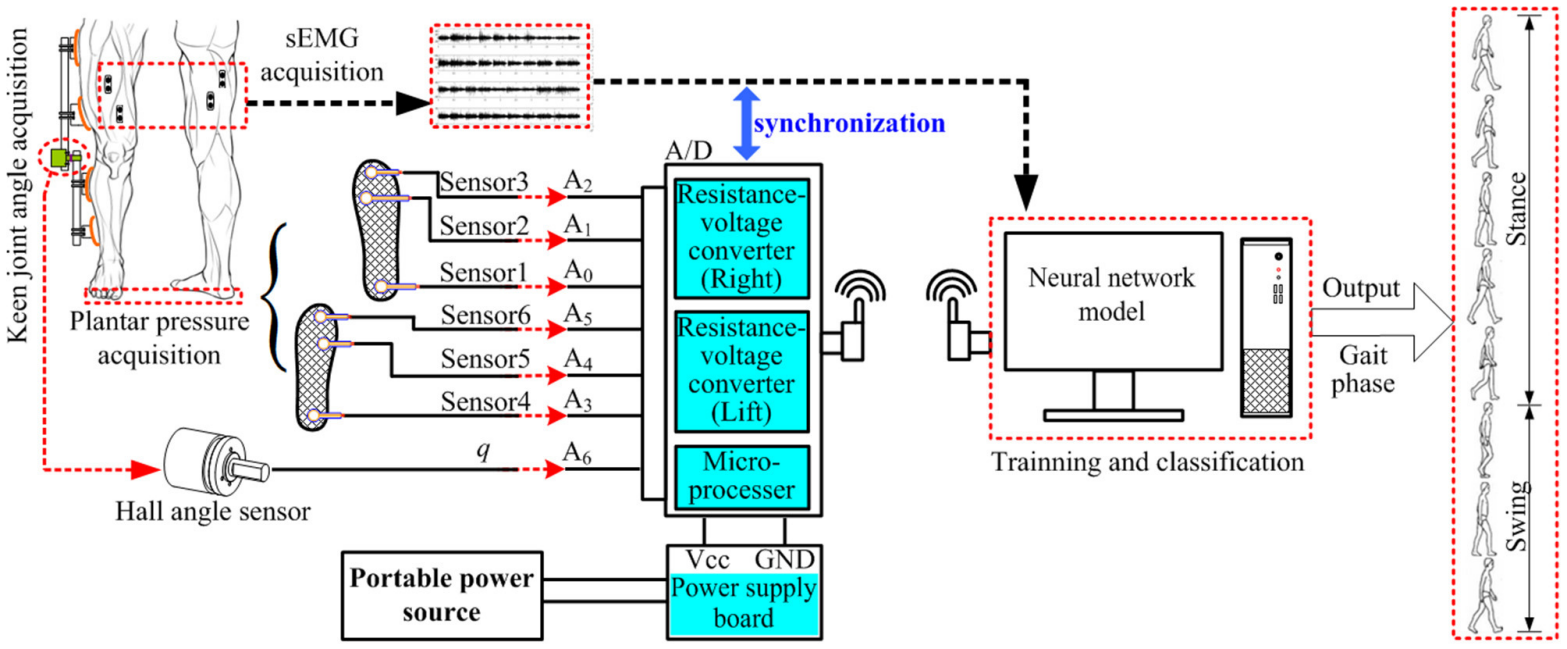
Structure of gait phase classification system.

Figure 1 shows the structure of the gait phase classification system. The sEMG acquisition system acquires the sEMG signal of the gait movement of the human leg, communicates wirelessly with the myoRESEARCH software in the computer, and transmits it to the neural network model of the computer. The Hall angle sensor acquires the knee joint angle information of human gait movement. The plantar pressure sensor collects the pressure information of the plantar in the stance phase of the human gait movement. The collected information is synchronized using a synchronization cable. The input to the neural network was the sEMG signal and the knee joint angle. After training with a label of gait events detected by plantar pressure information, it can output gait phase classification results in real time.

### Gait Information Acquisition

#### Plantar Pressure Acquisition Device

**Figure 2:**
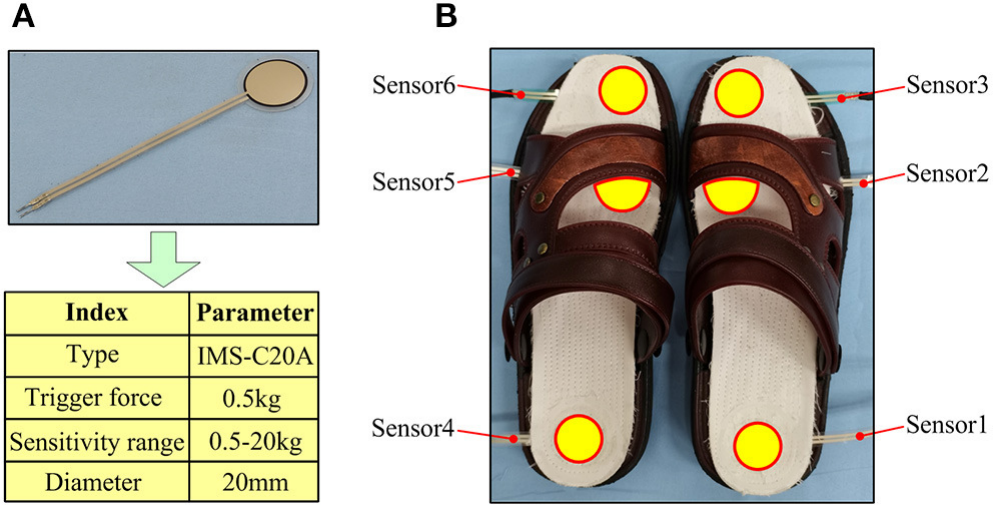
The composition of the plantar pressure collection device; **(A)** the thin-film pressure sensor and its parameters, **(B)** the physical product of the plantar pressure collection device.

The information collection of plantar pressure acquisition device is realized by a thin-film pressure sensor (IMS-C20A, Vicos Digital Tech. Ltd, China), which is, respectively, arranged in a multilayer cotton insole, as shown in Figure 2. Six identical thin-film pressure sensors are, respectively, placed on the heel, middle, and front of the two insoles to collect plantar pressure information on the heel, sole, and toe of the feet. When the sensor is being compressed, the amplified piezoelectric voltage is saved to the computer in the form of a digital signal through A/D. The specific parameters of the thin-film pressure sensor and the corresponding acquisition position relationship are shown in Table 1.

**Table 1:** Corresponding acquisition position of a thin-film pressure sensor.

**Thin-film pressure sensor**		**Position of acquirement**
**Left foot**	**Right foot**	
Sensor 6	Sensor 3	Toe
Sensor 5	Sensor 2	Sole
Sensor 4	Sensor 1	Heel

#### Knee Joint Angle Acquisition Device

**Figure 3:**
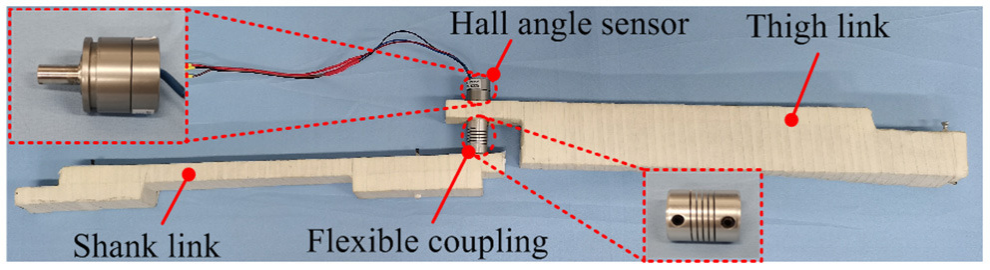
A knee joint angle acquisition device.

The knee joint angle acquisition device is composed of a Hall angle sensor (GT-B, Taizhou QT tech. Ltd, China), a 2-link, a flexible coupling, and several straps, as shown in Figure 3. The Hall angle sensor is a shaft-type angle measurement sensor. Its effective angle is 180 degrees, and the resolution is 0.18 degrees. The Hall angle sensor was installed on one end of the flexible coupling, which connects the shank and the thigh link. The function of the flexible coupling is to prevent the upper and lower links from being too rigid when the knee joint is moving, causing discomfort to the knee joint movement. The axis of the angle sensor was aligned with the human knee joint according to different individuals in order to fully synchronize the human leg and the knee joint.

#### sEMG Data Acquisition

**Figure 4:**
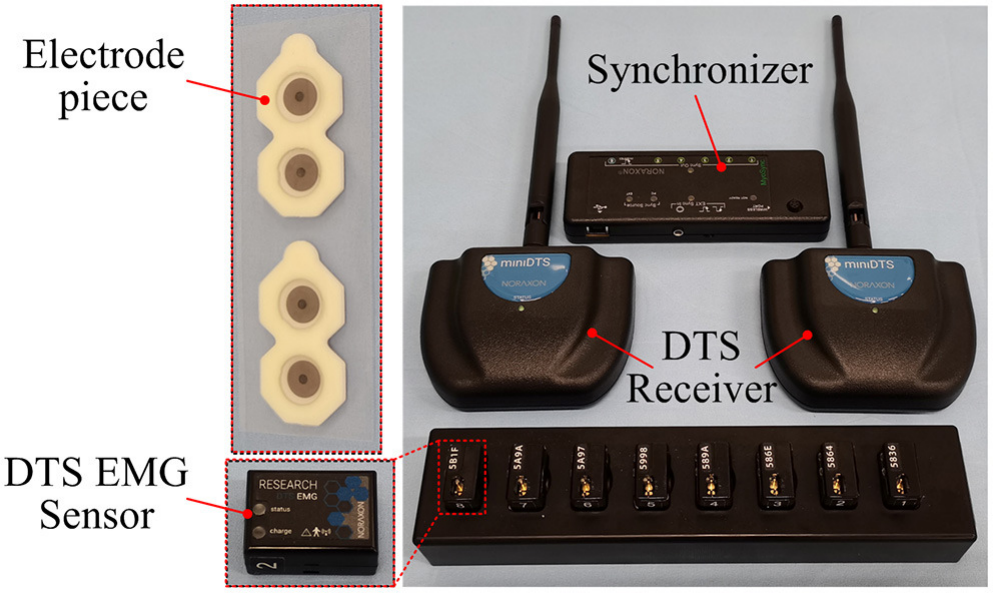
An sEMG acquisition device.

In the gait movement information acquisition experiment, the sEMG acquisition device used is an 8-channel Ultium-EMG sEMG signal acquisition instrument developed by Noraxon, USA, as shown in Figure 4. This device is a wireless transmission device that can acquire eight channels of sEMG signals with an acquisition frequency of up to 1,500 Hz. The timestamp of acquiring sEMG signals can be recorded simultaneously. The whole system includes eight sEMG signal sensors to obtain the sEMG signal of the human body, two receivers (Mini DTS Receiver) to transmit the acquired sEMG signal, a synchronizer to synchronize receiver data, and a sensor charger to turn on and off the sensor and charge the sensor. Combined with the myoMUSCLE software platform provided by the company, the sEMG signal can be acquired and simply processed in real time.

### Data Preprocessing

#### The Signal Denoising Method

The human sEMG signal is complex and feeble, and it is susceptible to the influence of many external factors, such as the signal acquisition device, the experimental environment, and the physical condition of the subject, resulting in the acquired sEMG signal containing a lot of external noise. During the experiment, the main noise of the sEMG signal is especially the power frequency interference and motion artifacts. Butterworth filter has the characteristics of a flat frequency response curve in the passband, no fluctuation, frequency response gradually drops to 0 in the stopband, and a steeper frequency response decline curve, which is often used for noise reduction of the sEMG signal (Gui et al., 2019; Li Z. et al., 2020; Ma et al., 2020). Therefore, to retain the useful data information in the collected sEMG signal and eliminate the interference noise during the experiment, the 20–450 Hz 4th-order Butterworth filter is used for filtering, and then the 50 Hz 2nd-order notch filter is used to eliminate the power frequency interference and can obtain effective sEMG data for the subsequent data analysis.

#### Data Set Construction

**Figure 5:**
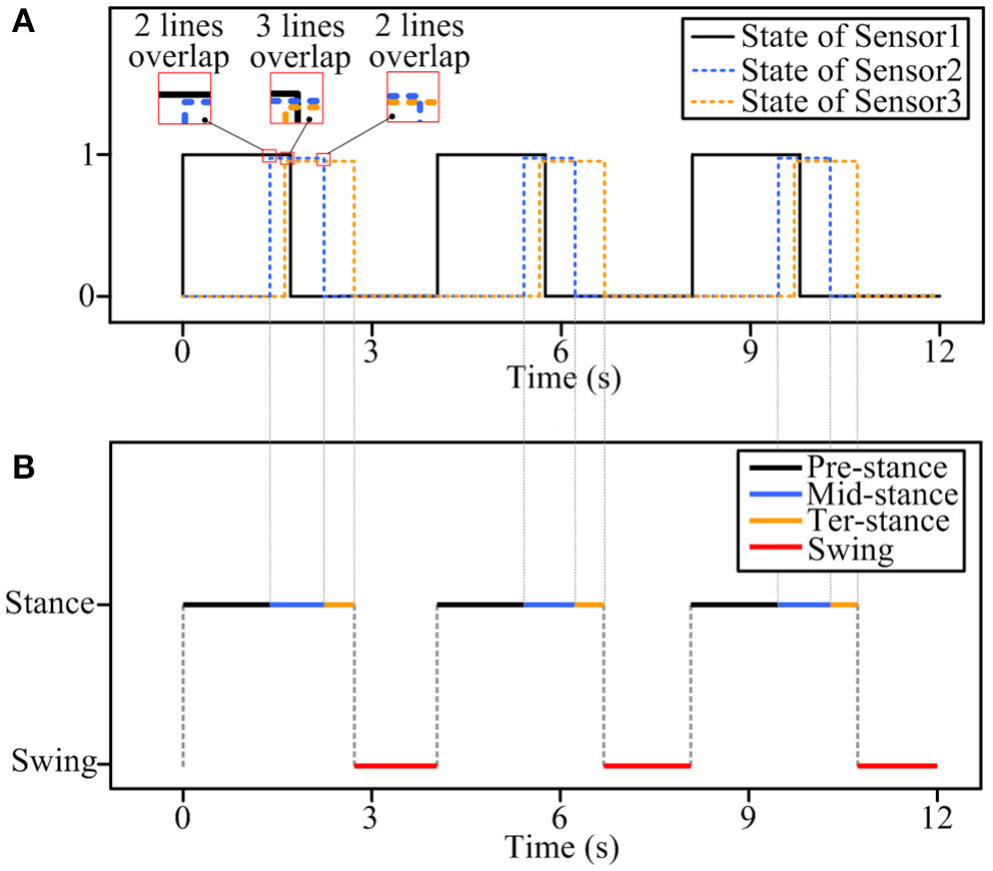
The gait phase classification process, **(A)** 0 and 1 states of the thin-film pressure sensor, **(B)** gait sub-phase classification.

The segmented sEMG with gait phase labels and knee joint angle data is required as input to the classification model. Because gait movement is the symmetrical movement of the left and right feet, the right foot is chosen as the research object. In order to simplify the segmentation process, three complete gait cycles are selected, and the acquired sEMG data are divided into gait phases according to the on-off state of the plantar pressure sensor. In the plantar pressure acquisition device, three thin-film pressure sensors measure the force between the heel, sole, and toe, and, according to the working state and working time of the three thin-film pressure sensors of the right foot, the gait is divided into four substages, namely, pre-stance, mid-stance, ter-stance, and swing phase. Figure 5 shows the classification process of the gait phase. In Figure 5A, the sensor is working when it is under pressure, which is represented by “1,” and when it is not under pressure, it is represented by “0.” In Figure 5B, red, black, blue, and orange correspond to the swing phase, the pre-stance phase, the mid-stance phase, and the ter-stance phase, respectively. The states of 0 and 1 of the thin-film pressure sensors reflect different gait substages at different times. When the thin-film pressure sensors sensor1 (4), sensor2 (5), and sensor3 (6) are all “0,” the gait phase is in the swing phase. When sensor1 (4) is “1” and sensor2 (5) and sensor3 (6) are “0,” it means that the heel touches the ground, the sole and toe do not, and the gait phase is in the pre-stance phase. When more than two of the sensor1 (4), sensor2 (5), and sensor3 (6) in the thin-film pressure sensor are “1,” and the thin-film pressure sensor corresponding to the sole remains “1,” more than two lines appear to overlap on the image. It shows that there are three situations: heel and sole contact the ground at the same time, but toe does not contact, or heel, sole, and toe contact the ground at the same time, or heel does not contact, sole and toe contact the ground at the same time, at this time, the gait phase is in the mid-stance phase. It is worth noting that, in practice, there is still a state, that is, sensor1 (4) and sensor3 (6) are not working, sensor2 (5) is working, and it is also in the mid-stance phase. When sensor1 and sensor2 (5) are “0” and sensor3 (6) is “1,” it means that only the toe of the foot contacts the ground, and the gait phase is at the ter-stance phase. Table 2 shows the corresponding relationship between the working state of the thin-film pressure sensor of the right foot and each substage of the gait phase. After the gait classification is completed, gait data are generated. The gait data, plantar pressure, and joint angle data have the same length.

**Table 2:** The relationship between the gait phase and the state of the thin-film pressure sensor.

**Gait phase**	**Plantar pressure sensor**		
	**Sensor1(4)**	**Sensor2(5)**	**Sensor3(6)**
Swing	0	0	0
Pre-stance	1	0	0
Mid-stance	1	1	0
	1	1	1
	0	1	0
	0	1	1
Ter-stance	0	0	1

Select the classic machine learning neural network model support vector machine (SVM) (Li et al., 2015) and deep learning neural network model long short-term memory (LSTM) (Liu et al., 2018) and back-propagation neural network (BPNN) (Chen et al., 2018) to compare with CNN. After filtering the sEMG data, use the sliding window to extract the mean absolute value (MAV) and root mean square (RMS) features of the sEMG signal, which can be expressed as:


(1) $$\begin{array}{l} MAV\;=\;\frac{\text{1}}{N}\overset{n}{\underset{i=1}{\sum }}|x_{i}| \end{array}$$

where *N* is the number of sample points in the sampling window, *x*_*i*_ is the amplitude of the *i*-th sEMG sample point.


(2) $$\begin{array}{l} RMS\;=\;\sqrt{\frac{1}{N}\overset{N}{\underset{i=1}{\sum }}{\left(x_{i}-\bar{x}\right)}^{2}} \end{array}$$

where *N* is the number of sample points in the sampling window, *x*_*i*_ is the amplitude of the *i*-th sEMG sample point, $\overset{-}{x}$ represents the average value of sEMG data in this window.

**Figure 6:**
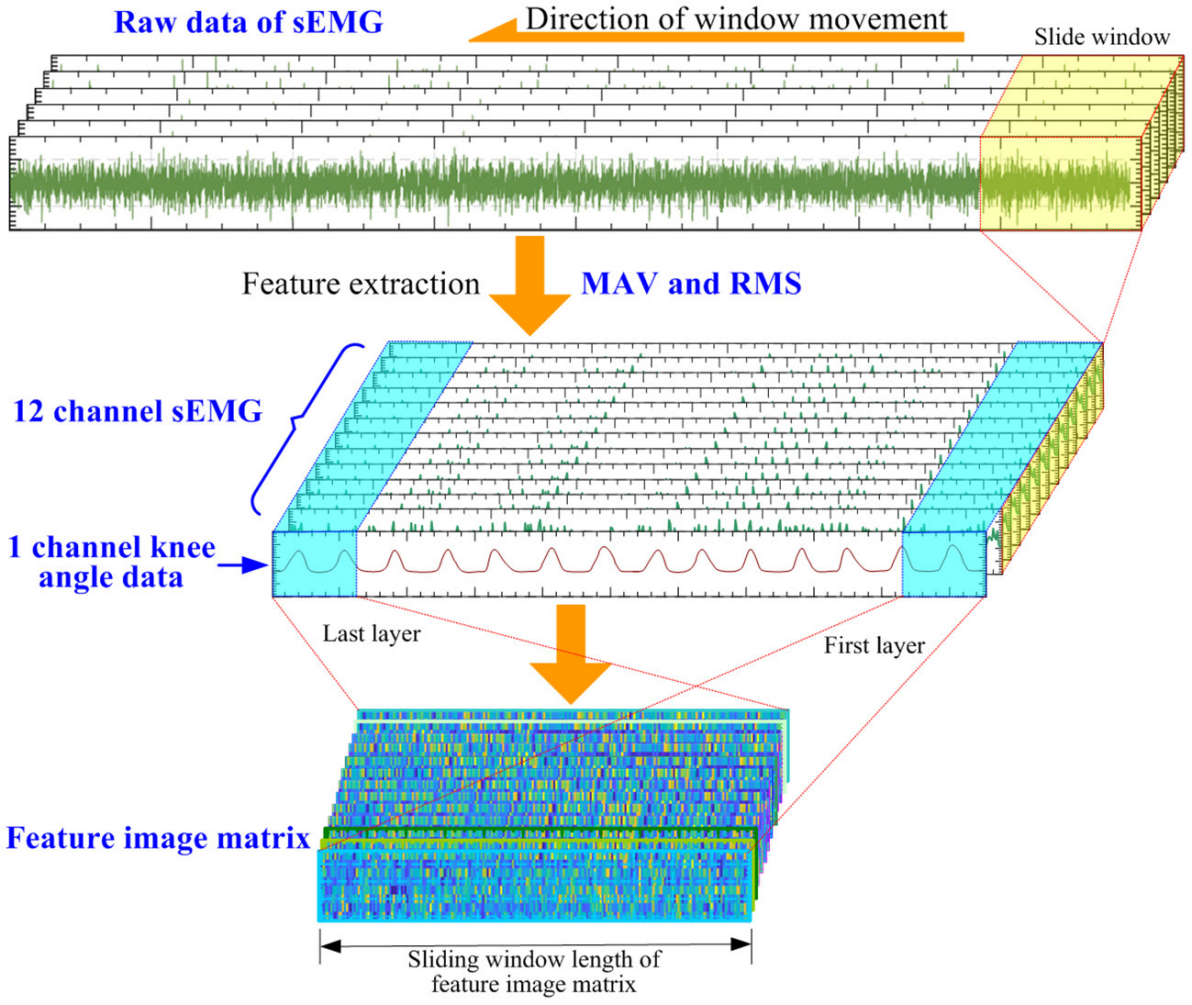
The process of data processing.

In this article, six-channel sEMG data of human lower limbs and one channel knee joint angle data are acquired. The sliding window method is used to extract RMS and MAV features from the raw sEMG data output by the sEMG sensor. The number of sample points in the sliding window is 30, and the sliding step length is 30. The sEMG feature data can be obtained after feature extraction processing of the sEMG data. At this time, the original sEMG data of each channel will generate two-channel (RMS and MAV) sEMG feature data, and the number of sEMG feature data channels will be changed from the 6 channels to 12 channels. At the same time, the length of sEMG feature data, the length of knee joint angle data, and the length of gait data are the same. Figure 6 shows the process of data processing.

Since the length of the sEMG feature data is the same as the length of the knee joint angle data, after the feature extraction process is completed, the knee joint angle data and the sEMG feature data are converted into an input feature image matrix. The number of sEMG feature data channels is 12, which, together with one channel of knee joint angle data, forms a 13-dimensional input feature image matrix. The input feature image matrix is slidingly intercepted by the sliding window method, and the length of the sliding window size is set to 20; therefore, the feature image matrix size is 20 × 13 × 1, and then is input into the neural network model. The knee joint angle data and feature data of the sEMG data are used as input. The total sample of sEMG data is 22,500, of which the first 80% is allocated as the training set and the last 20% as the test set.

Since the length of the sEMG feature data is the same as the length of the gait data, the time stamp corresponding to each sample point in the gait data and the sEMG feature data is consistent. When using the sliding window method to intercept the sEMG feature image matrix, the gait data corresponding to the sample points at the end of the sEMG feature image matrix is used as the label of the sEMG feature image matrix.

At this point, the training and testing data sets input to the neural network can be obtained. The neural network input data in the data set include the original sEMG signal after feature processing data and the knee joint angle data of the lower limbs, and the gait data as the label data in the data set. The sEMG signal data and the lower limb joint angle data are features fused through the convolutional neural network (CNN) to realize the gait classification.

### A Neural Network Model for Gait Phase Classification

**Figure 7:**
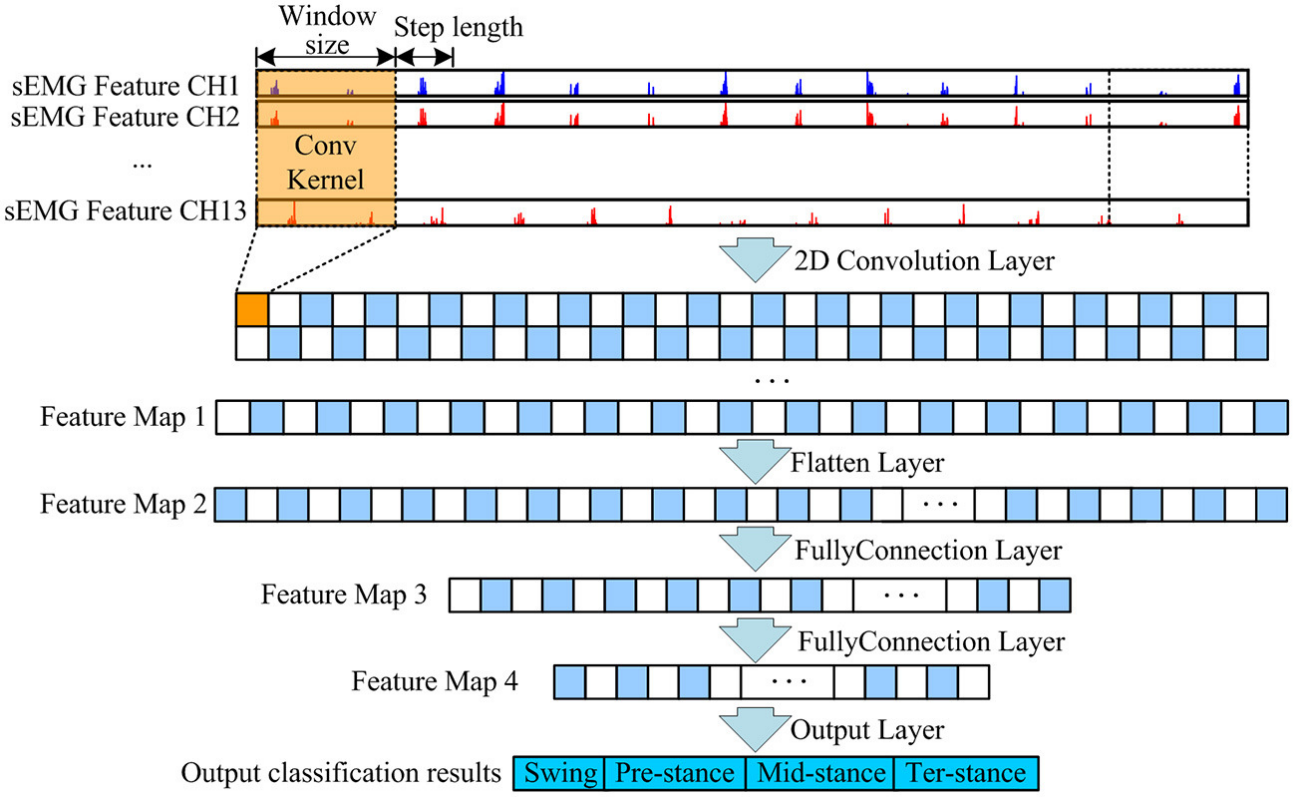
CNN architecture of the gait classification model.

Convolutional Neural Network is a feed forward neural network (Chen et al., 2019) and is the most commonly used network model in the field of deep learning (Zhai et al., 2017). Figure 7 shows the architecture of a CNN for gait phase classification. In this article, the dimension of the input data into the neural network model CNN is low, and the input data will be lost after adding the dimension reduction operation of the pooling layer, so the pooling layer is removed from the CNN model, and only the convolution layer exists. This will not affect the function of the CNN model and make its structure more concise. It also improves the training speed of the CNN model and the output speed of the gait phase classification results.

The model super parameter epochs are set to 300, the batch size is set to 100, softmax function is used as the activation function of the last layer of the model, Adam optimizer is used to update the model parameters, and the initial learning rate is set to 0.001. During training, the cross-entropy loss function is used to optimize the output. Softmax function and cross-entropy loss function are shown in formula (3) and formula (4)


(3) $$\begin{array}{l} {\overset{⌢}{y}}_{i}\;=\;softmax\left(x_{i}\right)=\frac{\exp\left(x_{i}\right)}{\overset{m}{\underset{j=1}{\sum }}\exp\left(x_{j}\right)},\;i=1,2,...,m \end{array}$$

(4) $$\begin{array}{l} J=-\frac{1}{N}\overset{N}{\underset{i=1}{\sum }}y_{i}\log\left({\overset{⌢}{y}}_{i}\right) \end{array}$$

where *x*_*i*_ is the input value of each node in the output layer, ${\overset{⌢}{y}}_{i}$ is the probability of actual output,*y*_*i*_ is the category label, *m* is the number of categories, and *N* is the number of batches input to the model at one time.

## Experiment

### Experimental Design

Five able-bodied subjects took part in this experimental study, aged between 24 and 28, height between 168 cm and 185 cm, and weight between 60 and 70 kg, and had not taken any strenuous exercise before the experiment. Before the experiment, to ensure that the experiment is effective, the following steps should be carried out:

- Clean the skin: remove the body hair on the tested muscle and wipe it with medical alcohol.
- Equipment placement: paste the electrode piece at the position of the muscle to be measured, and paste the electrode piece along with the muscle fiber of the leg, which is conducive to signal acquisition. Place the sEMG sensor about 2 cm away from the electrode and connect the electrode correctly. The electrode piece and the sEMG sensor are fixed with medical adhesive tape to prevent falling off during movement.
- Equipment detection: check the paste of the electrode sheet to ensure the paste is tight. Start the sEMG acquisition device; check the transmission status of each channel to ensure the normal transmission of the sEMG signal.

**Figure 8:**
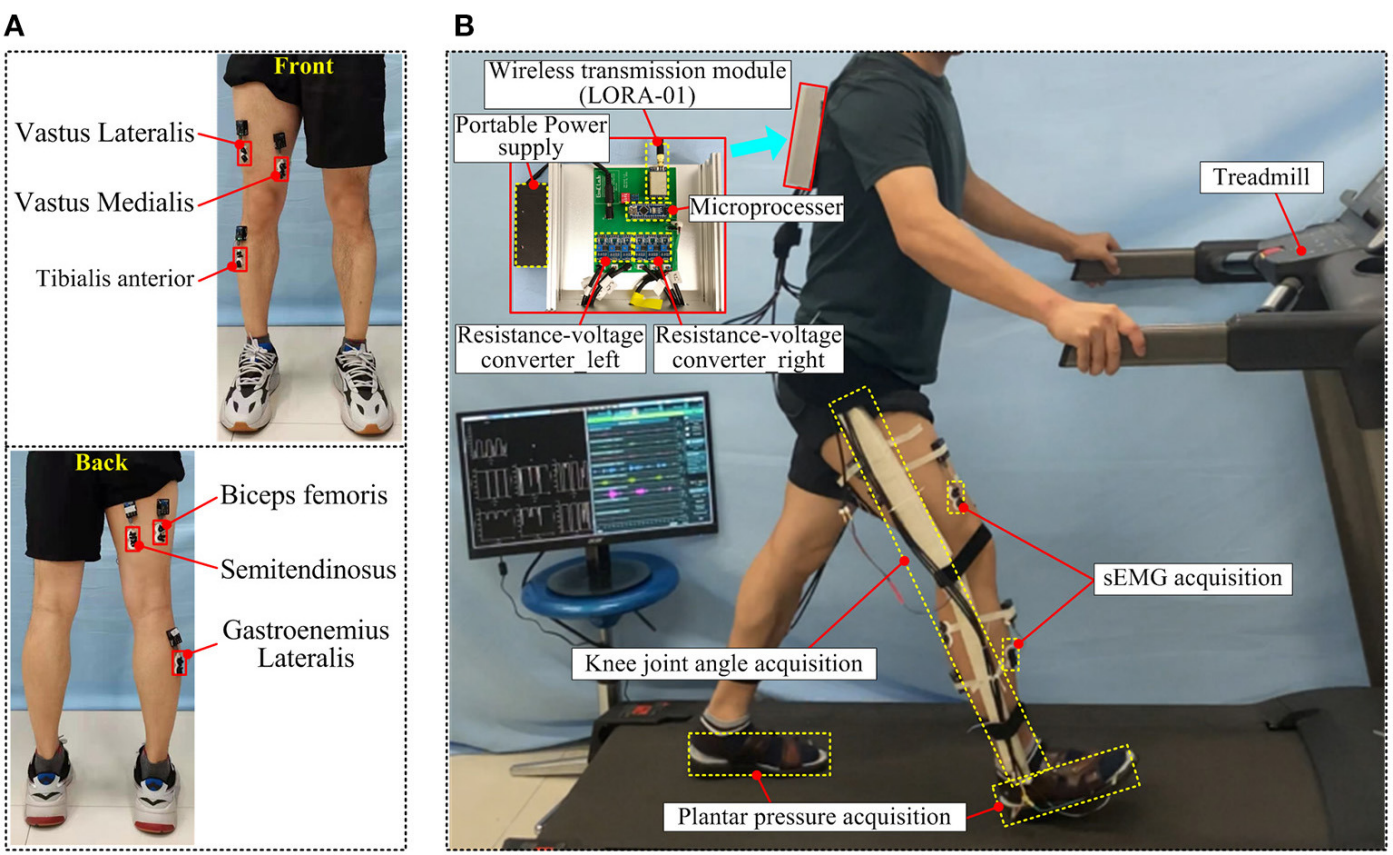
The gait information acquisition device, **(A)** the installation position of the sEMG sensor, **(B)** the wearing effect and information acquisition process of the gait information acquisition device.

Six sEMG sensors are arranged in muscle positions: vastus medialis (VM), vastus lateralis (VL), semitendinosus (ST), biceps femoris (BF), tibialis anterior (TA), and gastrocnemius lateralis (GA). When the EMG sensor is installed, wear other gait information acquisition equipment. The knee angle acquisition device is arranged on the outside of the thigh with an adhesive bandage, and the position of the Hall angle sensor is on the same axis with the rotation center of the knee joint, to ensure that the thigh rod and leg rod will not affect the rotation of knee joint (motion interference) when they move with the leg. Three thin-film pressure sensors embedded in the front, middle, and back of the insole were used to acquire the pre-stance, mid-stance, and ter-stance of the gait phase. Figure 8 shows the gait information acquisition device. Each muscle position corresponds to an sEMG acquisition device channel, and the corresponding relationship between the muscle and sEMG sensor channel is shown in Table 3.

**Table 3:** Corresponding channels of the sEMG sensor and muscle.

**Muscle location**		**Channel**
Thigh	Vastus Medialis	1
	Vastus Lateralis	2
	Biceps Femoris	3
	Semitendinosus	4
Shank	GastroenemiusLateralis	5
	Tibialis anterior	6

#### Different Speeds

Five subjects were tested with different gait speeds. Taking into account the conditions of healthy people, lower limb dyskinesia, and the elderly, the walking experiments were carried out at 1, 2, and 3 km/h, respectively, and the gait data were collected at three speeds. Among them, the speed of 3 km/h is close to the daily gait speed of normal people, while the speed of 2 and 1 km/h is gradually lower than the daily gait speed. During the gait walking experiment, the subjects rest 15 min between each gait speed to ensure that the leg muscles are in a relaxed state, and muscle fatigue may cause the distortion of sEMG information and affect the classification results of the gait phase. Each subject was acquired three times of gait data at the same gait speed, and the subjects rest for 5 min in each gait data acquisition to ensure the relaxation of leg muscles and check whether the equipment is loose to avoid affecting the results of gait data acquisition.

#### Comparison of Multi-Information and Single Information

In order to verify the superiority of the proposed method, the comparative experiments of multi-information and single information were carried out at 1-, 2-, and 3-km/h gait speeds. Plantar pressure is the label of sEMG data. Multi-information is to collect sEMG data of legs and knee joint angle data at the same time and input them into the gait classification model at the same time to classify the gait phase. Single information only collects sEMG information of legs during gait movement and inputs it into the gait classification model to classify the gait phase. At the same time, the multi-information and single information are compared with four gait classification models (SVM, BPNN, LSTM, and CNN), and the classification performance of different classification models with multi-information and single information input is obtained.

#### Five-Fold Cross-Validation

Cross validation is a common method used to verify the performance of the model in the process of modeling (Jung, 2018). It divides the original data into the training set and the test set. First, the training set is used to train the model, and then the test set is used to test the trained model so as to evaluate the performance of the model. In this paper, the 5-fold cross validation method is used to evaluate the model. Data of each subject are divided into five subsets. Each time, any subset is taken as the test set and the rest as the training set. After that, five models can be obtained. Finally, the average accuracy of the test set is taken as the evaluation index of the subject under the 5-fold cross validation method.

### Software Environment

The neural network model CNN of gait phase classification used in this study is compiled on the deep learning network framework Keras 2.3.0. The Keras network framework is an open-source artificial neural network library written in Python language, which can be used as the advanced application program interface (API) of TensorFlow. In this article, the python libraries used include NumPy, Sklearn, SciPy, and Matplotlib. The whole model implementation process is implemented on Pycharm software, and the model training is completed on a computer with an independent GPU. The specific configuration of the computer is shown in Table 4.

**Table 4:** Computer configuration information.

**Index**	**Parameter**
Central Processing Unit (CPU)	Intel Core i5 4570
Graphics Processing Unit (GPU)	Nvidia GTX1070 8GB
Operating system	Windows10
Computer memory	DDR3 1600 16GB
Software environment	Python 3.7.6

## Result and Discussion

Accuracy is a key index of human gait classification (Gao et al., 2021). In this article, two evaluation indexes are used, accuracy and F1-score, which can be expressed as:


(5) $$\begin{array}{l} \text{Accuracy}=\frac{T_{P}+T_{N}}{T_{P}+T_{N}+F_{P}+F_{N}} \end{array}$$

(6) $$\begin{array}{l} \text{F1-score}=\frac{2T_{P}}{2T_{P}+F_{P}+F_{N}} \end{array}$$

where *T*_*P*_ indicates correctly identifying positive samples as positive, *F*_*N*_ indicates wrongly identifying positive samples as negative, *F*_*P*_ indicates wrongly identifying negative samples as positive, and *T*_*N*_ indicates correctly identifying negative samples as negative.

### Different Speeds

**Figure 9:**
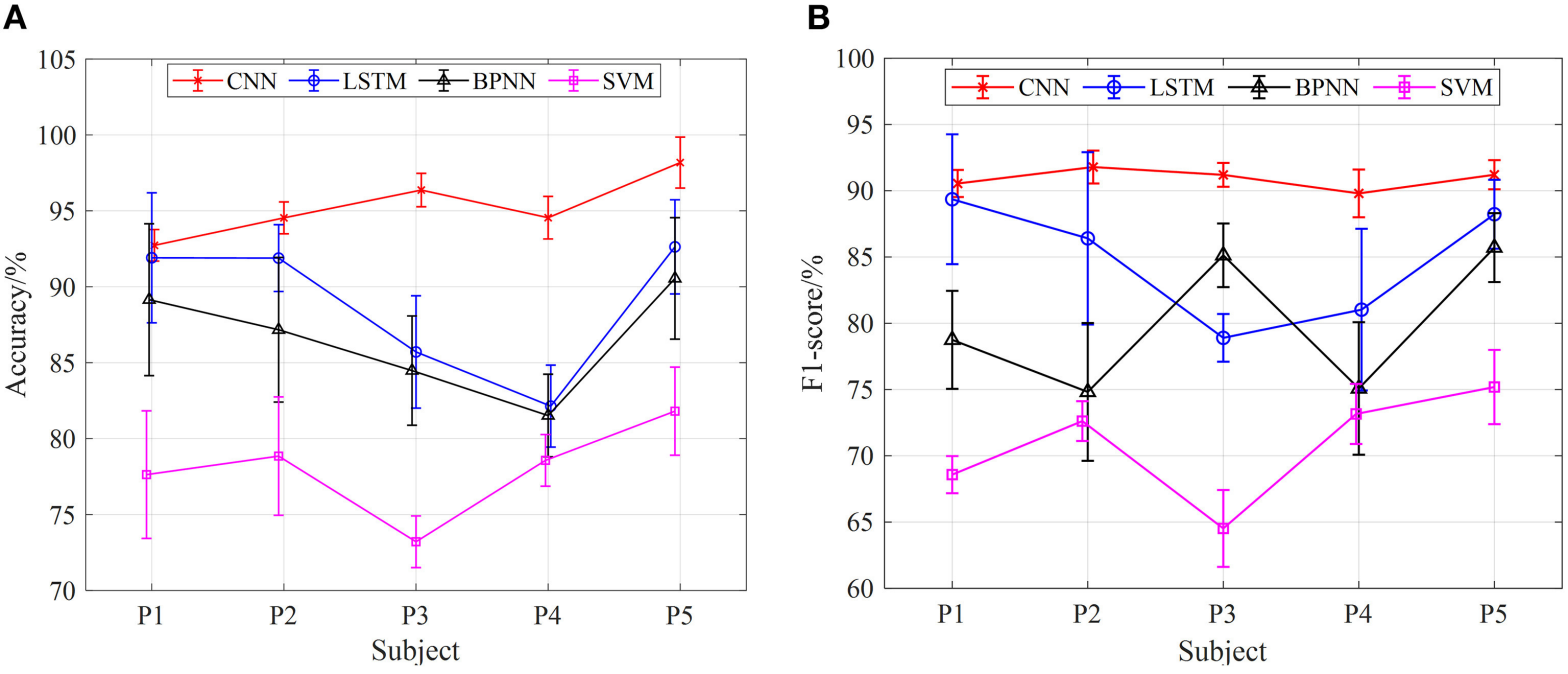
The results of gait phase classification of different models with the 5-fold cross-validation method at 1 km/h, **(A)** the average accuracy of gait phase classification of different models of five subjects, **(B)** the average F1-score of gait phase classification in different models of five subjects.

**Figure 10:**
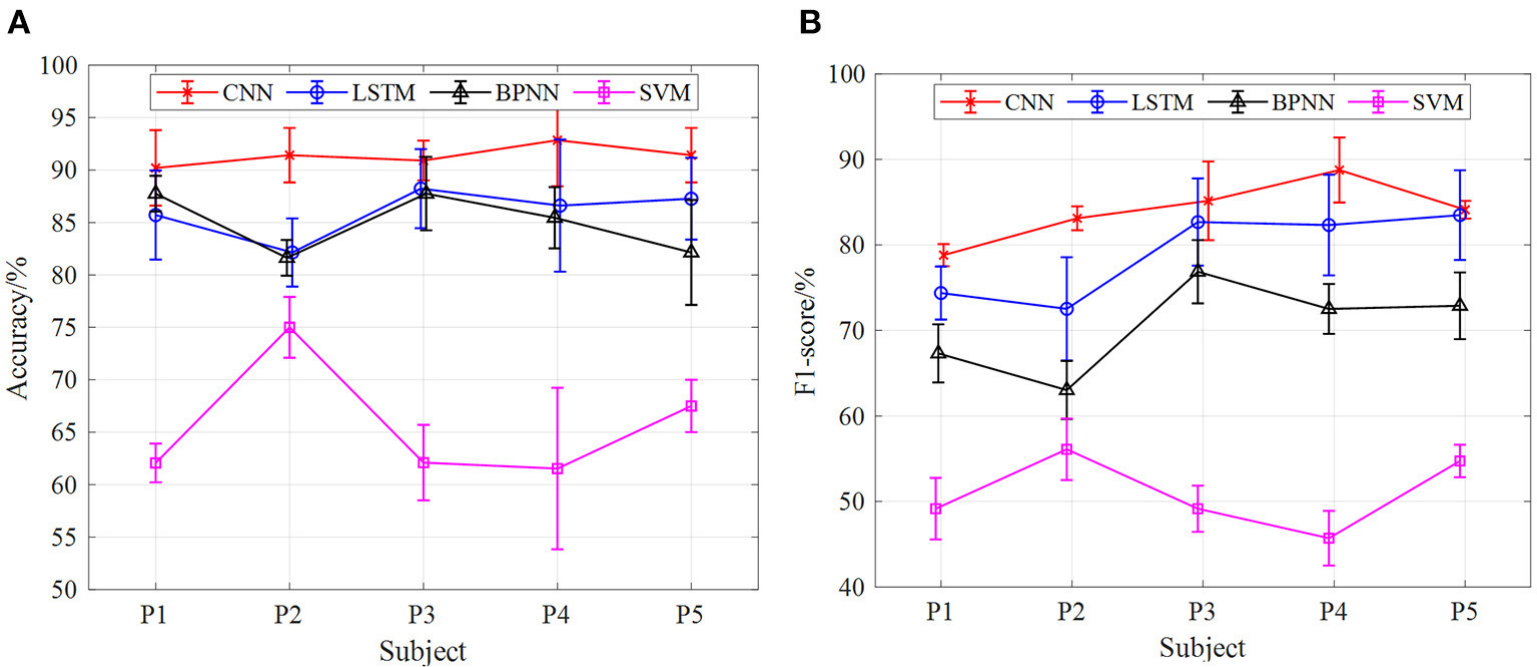
The results of gait phase classification of different models with the 5-fold cross-validation method at 3 km/h, **(A)** the average accuracy of gait phase classification of different models of five subjects, **(B)** the average F1-score of gait phase classification in different models of five subjects.

**Figure 11:**
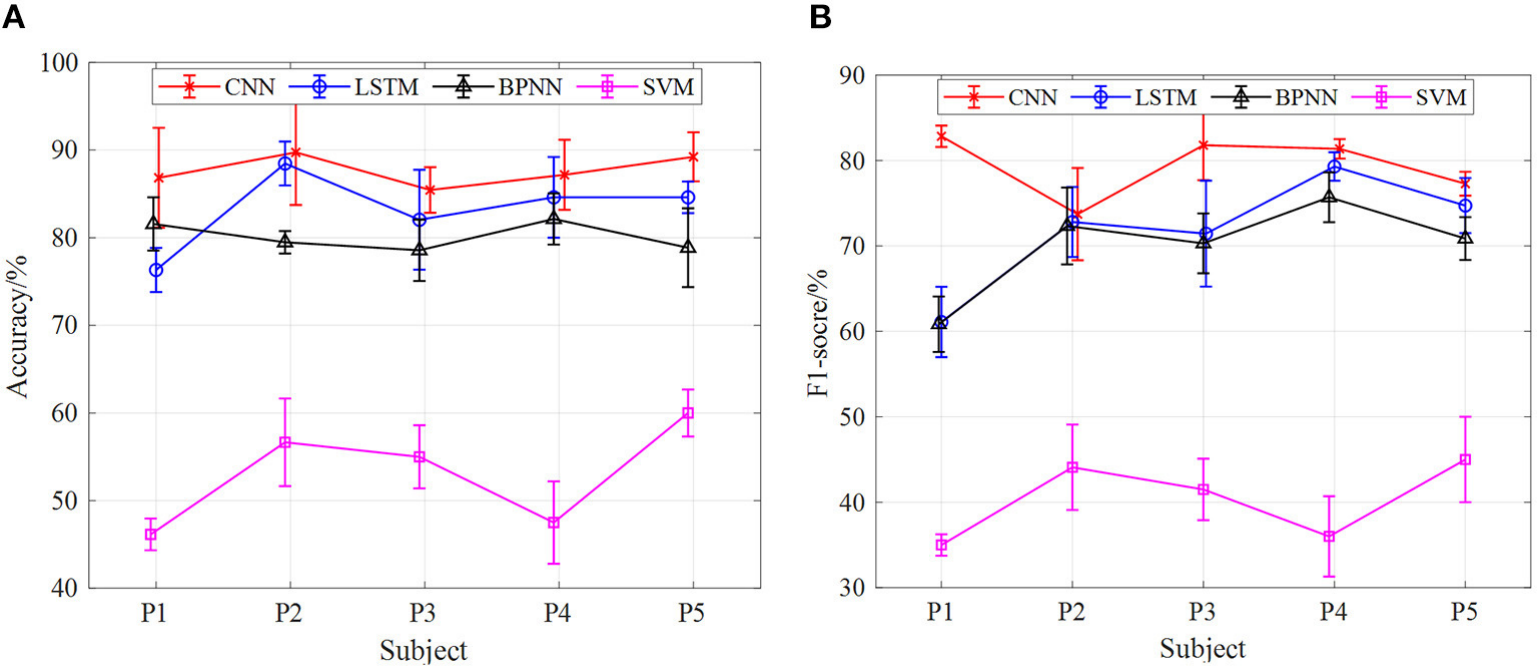
The results of gait phase classification of different models with the 5-fold cross-validation method at 3 km/h, **(A)** the average accuracy of gait phase classification of different models of five subjects, **(B)** the average F1-score of gait phase classification in different models of five subjects.

As shown in Figures 9–**11**, the classification results of four models (CNN, LSTM, BPNN, and SVM) of five subjects (P1, P2, P3, P4, and P5) with gait movement of 1 km/h, 2 km/h, and 3 km/h are shown. The classification accuracy and F1-score of the four models are different. In terms of Figure 9A, the classification effect of five subjects in the CNN model is the best, the classification accuracy of each subject is higher than that of the other three models, the prediction results of five subjects are between 93 and 98%, and the standard deviation of prediction results each subject is relatively small, indicating that the prediction results of the model are relatively stable. Compared with CNN, the classification effect of LSTM, BPNN, and SVM is unsatisfactory, the LSTM has the highest accuracy of 92% in five subjects, BPNN has the highest accuracy of 90% in five subjects, and SVM has the worst effect of 82%. In addition, the classification accuracy of LSTM, BPNN, and SVM in five subjects fluctuates greatly, and the standard deviation is also large, which indicates that the results of gait phase classification are unstable. Figure 9B shows five subjects in four neural network models F1-score. In the F1-score evaluation, the performance of CNN is better than the other three. The F1-score fluctuation of five subjects is relatively small, concentrated in 91–92%, and the standard deviation of the F1-score of each subject is also small, indicating a better classification effect. Compared with CNN, the other three models performed mediocrely in five subjects.

Figures 10, 11 show the classification results of the four models for five subjects at 2 and 3-km/h gait speeds. With the same trend of 1-km/h gait speed, the classification results of CNN are better than those of SVM, BPNN, and LSTM. However, with the increase of gait speed, the accuracy and F1-score of the four models are decreased. At 3 km/h, the classification accuracy of five subjects in the CNN model is about 90%, and the F1 score is up to 82%.

In terms of the analysis in Figures 9–11, we can see that, under the 5-fold cross-validation method, the CNN model outperformed the other three. The classification results of the five subjects are better than the other three models, and the classification effect is stable. It can well realize the classification of the gait phase, and the accuracy and F1-score have good performance, which proves the superiority and generalization ability of the CNN model. In addition, different gait speeds also have a great influence on the results of the gait phase classification of the four models. From the perspective of the four models as a whole, the accuracy of gait phase classification of the model is higher at lower gait speed, while the accuracy of gait phase classification of the model decreases significantly with the increase of gait speed. From Figures 9–11, when the gait speed is 1 km/h, the accuracy of CNN, LSTM, BPNN, and SVM is 98, 92.5, 90.5, and 82% respectively, and the highest value of F1-score is 92, 89.5, 85, and 75%, respectively; when the gait speed is 3 km/h, the accuracy of CNN, LSTM, BPNN, and SVM is 90, 89, 82, and 90%, respectively, and the highest value of F1-score is 82, 80, 76, and 45%, respectively.

**Figure 12:**
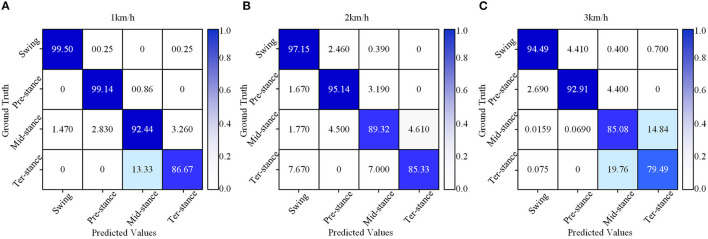
The accuracy confusion matrix of different gait speeds of the CNN model at the 5-fold cross-validation method, **(A)** a confusion matrix of 1 km/h, **(B)** a confusion matrix of 2 km/h, **(C)** a confusion matrix of 3 km/h.

Figure 12 shows the average confusion matrix of the accuracy of the three speeds of the best classification model CNN in the 5-fold cross-validation method. The confusion matrix provides visualization of the classification performance of the gait substages. The vertical axis of the matrix represents the real category of the test data set, and the horizontal axis of the matrix represents the corresponding classification results. The values of these three confusion matrices are the average classification accuracy of all objects under three different gait speeds. In terms of Figure 12, the gait phase substages of the three speeds have excellent classification results, and the gait phase substages of each speed can be divided. It can be seen from the figure that the classification accuracy of the swing and the pre-stance is above 99.14 and 92.91%, respectively, under the three speeds. Especially when the speed is 1 km/h, the classification accuracy reaches the highest, 99.50 and 99.14%, respectively. In the mid-stance phase and the ter-stance phase, the classification accuracy performance is undistinguished. Compared with the swing phase and the pre-stance phase, the highest classification accuracy at 1 km/h is only 92.44 and 86.67%, and the highest classification accuracy at 2 km/h is 89.32 and 85.33%, which dropped by 3.12 and 1.34%, respectively; when the gait speed is 3 km/h, the classification accuracy is 85.08 and 79.49%, which dropped by 7.36 and 7.18%, respectively.

In terms of Figure 12, gait speed has a great influence on the classification of gait phases. At a gait speed of 1 km/h, the classification effect of the gait phases is better, and the classification accuracy of the substage of the gait phases is high, especially for the swing phase. When the gait speed is 3 km/h, the classification effect of the substage of the gait phase is lower than 1 km/h, and the accuracy also gradually decreases. For the mid-stance and ter-stance, the classification accuracy of each speed is significantly lower than the swing and pre-stance, and the main reason is that the number of samples acquired during the movement is insufficient, which leads to the classification effect becoming mediocre.

### Comparison of Multi-Information and Single Information

**Figure 13:**
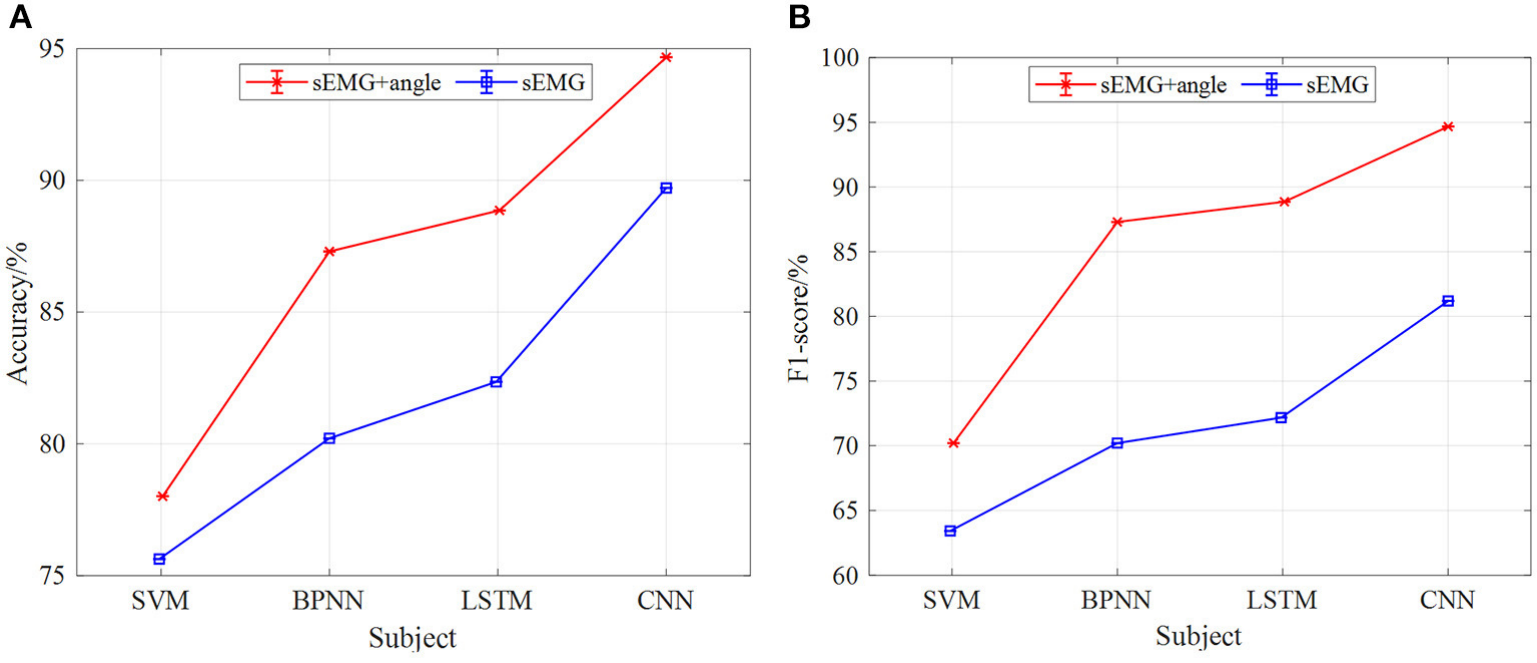
The comparison results of different models of multi-information and single information input with the 5-fold cross-validation method at 1 km/h, **(A)** comparison results of four model accuracy with different input information, **(B)** comparison results of four models F1-score with different input information.

**Figure 14:**
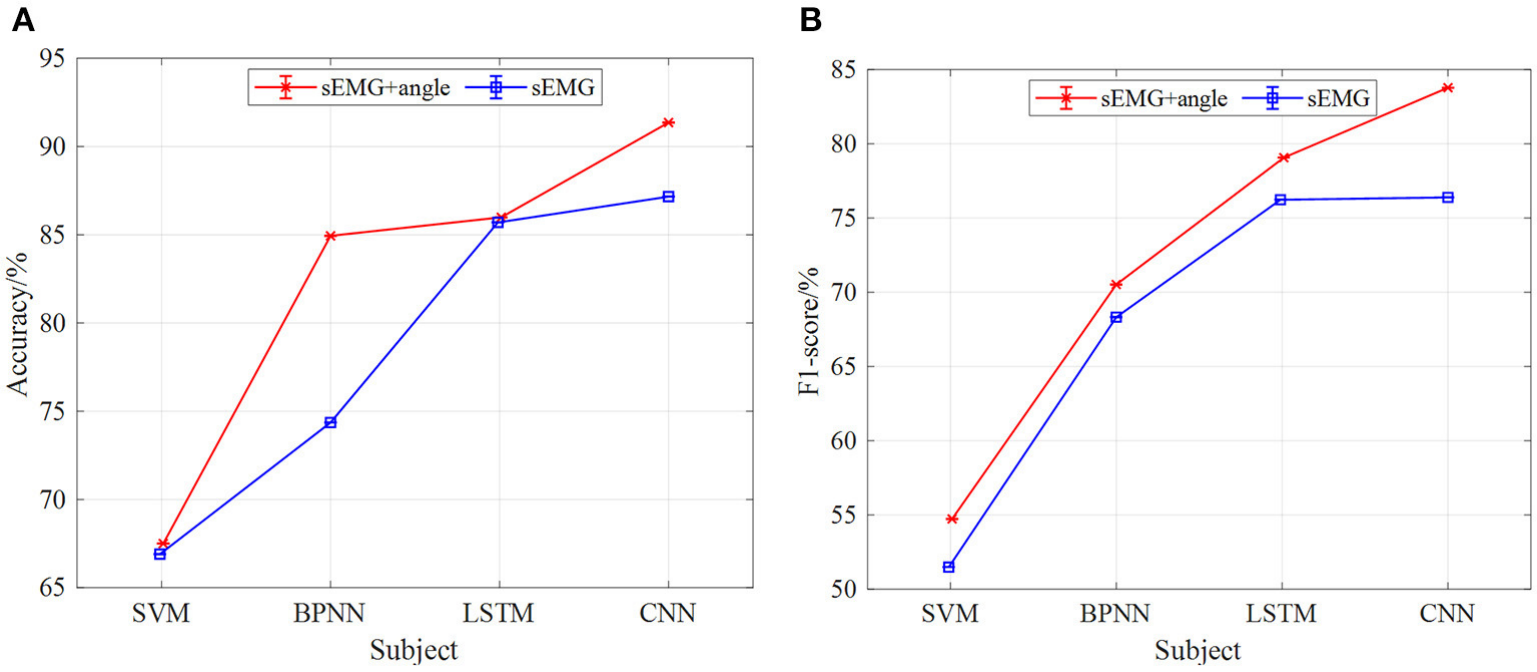
The comparison results of different models of multi-information and single information input with the 5-fold cross-validation method at 2 km/h, **(A)** comparison results of four model accuracy with different input information, **(B)** comparison results of four models F1-score with different input information.

**Figure 15:**
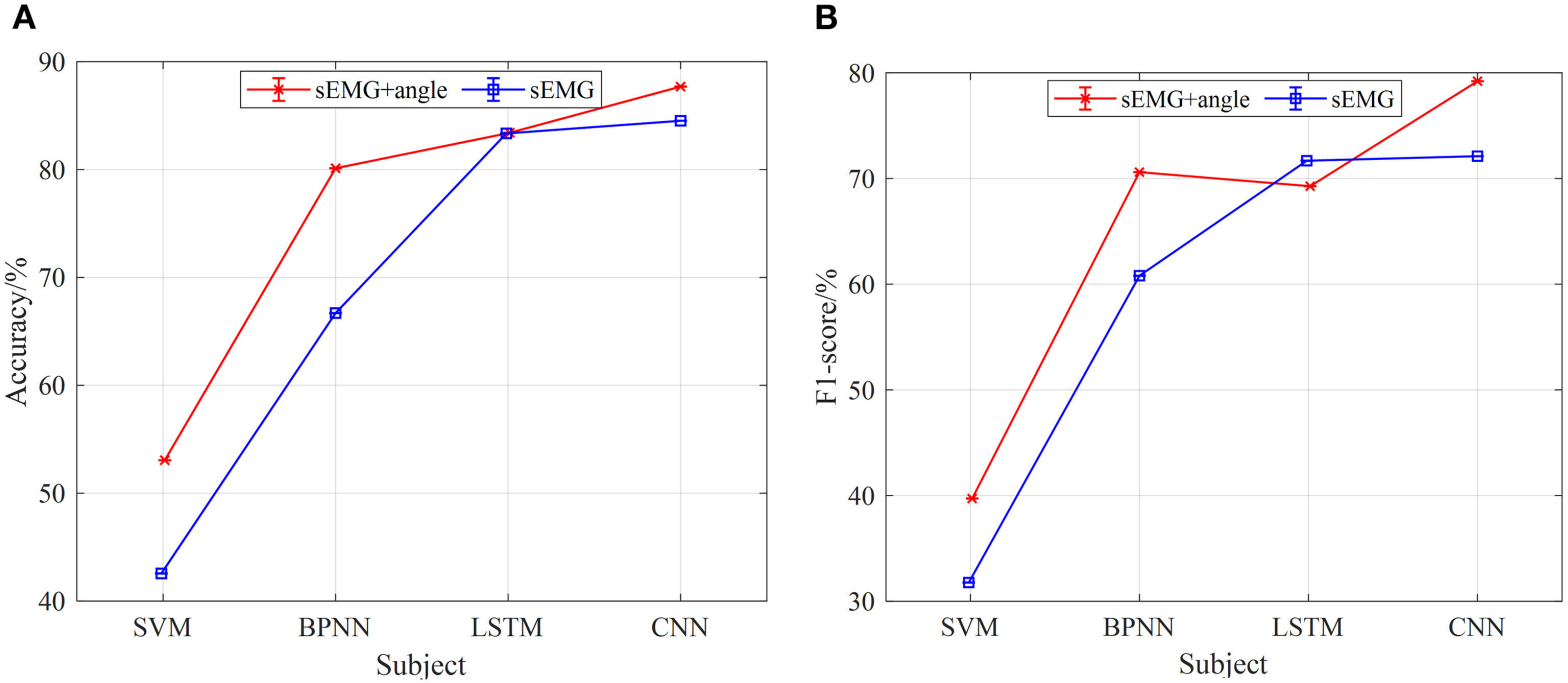
The comparison results of different models of multi-information and single information input with the 5-fold cross-validation method at 3 km/h, **(A)** comparison results of four model accuracy with different input information, **(B)** comparison results of four models F1-score with different input information.

In addition, under the model of the 5-fold cross-validation method, we carried out comparative experiments of different input signals to the neural network model. At the same speed, the accuracy of gait phase classification and the average value of F1-score of five subjects (P1, P2, P3, P4, and P5) were taken to compare the classification effect of sEMG and sEMG + angle input to SVM, BPNN, LSTM, and CNN neural network models. Figures 13–**15** show the gait classification results of different input signals in the four models under three motion speeds of 1, 2, and 3 km. In terms of Figure 13A, when sEMG + angle is used as input, the result of gait phase classification is better than that of sEMG alone, and the classification accuracy of CNN is the highest, and the average accuracy of five subjects is close to 95%. The second was LSTM. When sEMG + angle was used as input, the average accuracy of five subjects was close to 89%. BPNN is worse than CNN and LSTM. When sEMG + angle is used as input, the average accuracy of five subjects is about 87.5%, and the SVM classification effect is the worst, about 78%. When sEMG was used as input alone, the average accuracy of five subjects in the CNN model was close to 90%, and the accuracy of LSTM, BPNN, and SVM was about 82.5, 80.5, and 76%, respectively. It can be seen from the figure that, when sEMG + angle is used as input, compared with sEMG alone, the classification accuracy of SVM, BPNN, LSTM, and CNN is improved by 2.6, 8, 7.3, and 5.6%, respectively. At the same time, F1-score is used as the evaluation index. As shown in Figure 13B, when sEMG + angle is used as the input of the neural network model, the classification result is better than that of sEMG alone. As before, taking the average value of F1 score of five subjects, the average value of F1-score of SVM, BPNN, LSTM, and CNN neural network models reaches 70, 87.5, 89, and 95%, respectively, which is 10, 20, 18.5, and 14.7% higher than that of sEMG alone.

Figures 14, 15 are the comparison results of 2- and 3-km/h gait speed, respectively, and the overall trend is the same as Figure 13. The classification accuracy and F1-score of the four neural network models are gradually increasing from SVM to CNN, and the classification results with sEMG + angle as input are better than those with sEMG as input alone. It is worth noting that when the gait speed is 3 km/h, the F1-score of LSTM is higher than sEMG + angle, which is different from the results in Figures 13, 14. The reason for this phenomenon is that, among the five subjects, the effect of information collection is not good due to the fast gait speed or unstable walking posture; when data are input into the neural network, the output F1-score is low, resulting in low average F1-score. In general, when sEMG + angle is used as input, the output results of four neural network models are better than that of sEMG alone. Therefore, multi-information has a satisfactory classification effect for gait phase classification.

Table 5 shows the classification time of several classification models on a single sample and the delay time of each sensor of the gait acquisition device. In the gait information acquisition experiment, the delay time of the sEMG sensor is 114 ± 5 ms, and the delay time of the sensor of the knee joint angle acquisition device is about 0.1 ± 0.03 ms, and the delay time of the sensor of plantar pressure acquisition device is about 0.12 ± 0.05 ms. At the same time, in the process of gait phase model classification, the single sample classification time of SVM, BPNN, LSTM, and CNN is 0.18, 0.33, 1.53, and 0.37 ms, respectively. Because sEMG information acquisition, knee angle information acquisition, and plantar pressure information acquisition are carried out at the same time, the delay time of the sEMG sensor includes the sensor delay time of the knee joint angle acquisition device and the plantar pressure acquisition device. It is known from Li K. et al. (2020), the delay time of human sEMG action is about 150 ms, and the delay time of the sEMG sensor and the model for single sample classification is <150 ms, so the gait phase classification system has real-time performance. Besides, although the classification time of CNN is slightly higher than SVM and BPNN in a single sample, the classification effect of CNN is better than SVM and BPNN, so CNN is selected as the neural network model of gait phase classification.

**Table 5:** Sensor delay time and classification time of a single sample.

**Index**	**SVM**	**LSTM**	**BPNN**	**CNN**
sEMGsensor delay time	114 ± 5 ms	114 ± 5 ms	114 ± 5 ms	114 ± 5 ms
Keen angle acquisition sensor delay time	0.1 ± 0.03 ms	0.1 ± 0.03 ms	0.1 ± 0.03 ms	0.1 ± 0.03 ms
Plantar pressure acquisition sensor delay time	0.12 ± 0.05 ms	0.12 ± 0.05 ms	0.12 ± 0.05 ms	0.12 ± 0.05 ms
Classification time of single sample	0.18 ms	0.33 ms	1.53 ms	0.37 ms

## Conclusion

In this article, a gait phase classification method based on multi-information fusion is proposed. The principle of the gait phase classification system, the structure of the gait information acquisition device, and the data preprocessing method are given. The performance and the generalization ability of the model are proved by 5-fold cross-validation. Two task experiments using the proposed multi-information fusion gait phase classification method were carried out. In the 5-fold cross-validation method, the experimental results demonstrated that the average accuracy and the average F1-score of the proposed method reach 98 and 92%, respectively, for the gait phase classification at 1-km/h gait speed. For different input information experiments, in the case of three gait speeds, the classification effect of multi-information is far better than that of single information. At the same time, the delay time of the sEMG sensor, the Hall sensor of knee angle acquisition device, and the thin-film pressure sensor of plantar pressure acquisition device was measured, and the time of the neural network model to classify a single sample was also measured, which proved the real-time performance of gait phase classification system. Due to the integration of the information directly related to gait movement, the proposed multi-information fusion method for gait phase classification is better than the reported (Liu et al., 2016; Luo et al., 2020) gait classification method or system.

## Data Availability Statement

The original contributions presented in the study are included in the article/supplementary material, further inquiries can be directed to the corresponding author/s.

## Ethics Statement

The studies involving human participants were reviewed and approved by Medical Ethics Committee, Department of Medicine, Shenzhen University. The patients/participants provided their written informed consent to participate in this study.

## Author Contributions

YZ, GC, and AZ: methodology. YZ and ZL: software. YZ, ZL, SC, BH, and HC: performed data collection. GC: funding acquisition, project administration. YZ: analysis of the collected data and wrote the first draft of the manuscript. YZ and WL: writing-review and editing. All authors contributed to the article and approved the submitted version.

## Funding

This work was supported by the National Natural Science Foundation of China under Grant NSFC U1813212.

## Conflict of Interest

The authors declare that the research was conducted in the absence of any commercial or financial relationships that could be construed as a potential conflict of interest.

## Publisher's Note

All claims expressed in this article are solely those of the authors and do not necessarily represent those of their affiliated organizations, or those of the publisher, the editors and the reviewers. Any product that may be evaluated in this article, or claim that may be made by its manufacturer, is not guaranteed or endorsed by the publisher.
